# Mechanism of activation of TLR4/NF-κB/NLRP3 signaling pathway induced by heat stress disrupting the filtration barrier in broiler

**DOI:** 10.1186/s12917-024-04411-2

**Published:** 2024-12-28

**Authors:** Hui-li Dong, Xing-yue Wu, Fei-yao Wang, Hao-xiang Chen, Si-liang Feng, Chen-yang Zhou, Zhan-qin Zhao, Li-fang Si

**Affiliations:** College of Animal Science and Technology, Henan University of Science and Technology, Luoyang, 471000 China

**Keywords:** Heat stress, Broiler, Antioxidant capacity, Heat shock protein, Renal filtration barrier, TLR4/NF-κB/NLRP3

## Abstract

**Background:**

High-temperature environment can cause acute kidney injury affecting renal filtration function. To study the mechanism of renal injury caused by heat stress through activates TLR4/NF-κB/NLRP3 signaling pathway by disrupting the filtration barrier in broiler chickens. The temperature of broilers in the TN group was maintained at 23 ± 1 °C, and the HS group temperature was maintained at 35 ± 1℃ from the age of 21 days, and the high temperature was 10 h per day, and one broiler from each replicate group at the age of 35 and 42 days was selected for blood sampling, respectively.

**Results:**

The ELISA results demonstrated that in comparison to the TN group, serum CORT content of broilers in the HS group was all remarkably elevated (*P* < 0.01); the levels of IL-6 and TNF-α in the serum were remarkably elevated (*P* < 0.05 or *P* < 0.01); serum CAT and SOD activities were all remarkably reduced (*P* < 0.05 or *P* < 0.01), and serum LDH activity and MDA content were all remarkably decreased (*P* < 0.05); serum BUN and CRE levels were remarkably elevated (*P* < 0.01). Pathological sections and transmission electron microscopy demonstrated that the structure of the renal filtration barrier in the HS group damaged gradually with the prolongation of heat stress in comparison to the TN group, but the damage was reduced at 42 days of age; the levels of TLR4, MyD88, NF-κB, NF-κB-p65, NLRP3, caspase-1 and IL-1β mRNAs were all up-regulated (*P* < 0.05 or *P* < 0.01) in renal tissues of the HS group, indicating that heat stress caused damage to the morphological structure and function of the renal filtration barrier and that TLR4/NF-κB/NLRP3 pathway was also affected by heat stress, leading to increased activity (*P* < 0.05 or *P* < 0.01).

**Conclusions:**

It demonstrated that heat stress caused detrimental effects on both the morphological structure and function of the renal filtration barrier, and the initiation of the TLR4/NF-κB/NLRP3 signaling pathway exacerbated the inflammatory damage, leading to increased thermal damage to renal tissues and glomerular filtration barriers; however, with the prolongation of heat stress, broilers gradually developed heat tolerance, and the damage to the renal tissues and filtration barriers triggered by heat stress was mitigated.

## Background

Modern broilers mostly adopt large-scale and intensive breeding modes, which alleviates the impact of external environmental changes on broilers, but seasonal and sudden high temperatures also cause a series of problems. As poultry are covered with feathers, their sweat glands are underdeveloped and their self-regulation is poor, resulting in their inability to exceed temperatures beyond the range of the isothermal zone in a timely manner [1]. When the ambient temperature exceeds 30 °C, for every 1 °C rise, the feed intake of broilers will be reduced by 4.6%, growth will be slow, activity will be weakened, resistance will be reduced [2], and various diseases will be easily triggered, thus affecting the growth performance of broilers and meat quality [3–5]. When the ambient temperature exceeds the isothermal range of broilers (generally 18 ~ 26 °C), the body will produce some non-specific reactions, leading to an increase in reactive oxygen radicals, which can cause oxidative damage to several organs, and in severe cases, shock or death. Heat stress has been reported in many parts of the world to cause serious economic losses in the aquaculture industry [6].

Heat stress leads to serum hormone disorders, especially the significant elevation of serum corticosterone, which has become a hallmark indicator of heat stress [7], which is manifested by elevated levels of reactive oxygen species (ROS) in the brain and muscle of poultry, which in turn causes oxidative damage, resulting in altered tissue structure [8]. Under heat stress, the redox balance in the body of livestock and poultry is disrupted, generating excessive oxidative free radicals and reactive oxygen species, destroying the balance of the antioxidant enzyme system, and ultimately causing damage to tissues and cells [9]. Catalase (CAT) mainly decomposes hydrogen peroxide and reduces the production of oxygen free radicals; superoxide dismutase (SOD) is an important defense enzyme in the body, which can scavenge oxygen free radicals; malondialdehyde (MDA) is the end product of lipid peroxidation caused by oxygen free radicals, and its content is negatively correlated with the antioxidant capacity of the body [10]. Oxidative stress leads to excessive elevation of pro-inflammatory cytokines, including tumor necrosis factor-α (TNF-α), interleukin-6 (IL-6), and interleukin-1 beta (IL-1β), which triggers inflammatory responses [11, 12]. It has been suggested that TNF-α can activate T cells to produce a lot of inflammatory factors, which in turn contributes to the inflammatory response.IL-1β, as an important inflammatory cytokine, primarily contributes to numerous autoimmune diseases and influences cell proliferation, differentiation, and modulation of cell death, and also regulates the quantities of IL-6 and TNF-α [13].

Creatinine and urea nitrogen are indicators of serum renal function commonly used in clinical practice to assess renal status [14]. Urea nitrogen (BUN) is the end product of the metabolism of protein-like substances in biological organisms. Creatinine (CRE), a product of muscle metabolism, is metabolized mainly by the kidneys and excreted in the urine, which can reflect glomerular filtration. Under normal conditions, the production and excretion of urea nitrogen are in dynamic balance. Serum urea nitrogen and serum creatinine levels are significantly elevated only when renal function is severely impaired [15, 16].

Heat stress also causes the organism to undergo a heat shock response that triggers the overexpression of heat shock proteins. Heat shock protein 60 (HSP60) is a typical mitochondrial protein in eukaryotes and is involved in the conformational correction of proteins after misfolding [17]. Heat shock protein 70 (HSP70) restricts pro-inflammatory gene nuclear factor-kappa B (NF-κB) signaling by inhibiting NF-κB-p65 translocation to the nucleus. Heat shock protein 90 (HSP90) is distributed in glomerular capillary endothelial cells and their basement membranes and plays an important role in labeling renal injury. The study found that transportation stress resulted in elevated HSP90 in rat kidneys, indicating kidney damage [18].

The glomerulus is a filtration unit of the kidney that is used to excrete unwanted chemicals and waste products from the blood and is essential for maintaining the stability of the internal environment of the body. Inside the glomerulus, there exists a fine capillary wall structure which can be divided into three layers, the most crucial of which is the basement membrane, which is located in the middle layer. On the inner side of the basement membrane is covered with a layer of endothelial cells; on the outer side are the epithelial cells of the dirty layer, also known as foot cells. The foot cells are rich in cytoplasm and form elongated projections called peduncles, which are tightly attached to the outer loose layer of the basement membrane. The tiny spaces between the pedicles are called filtration slits, and there is a membrane between adjacent pedicles called the filtration slit. Together, the endothelial cells, basement membrane, and podocytes build an important physiological structure, the glomerular filtration barrier (GFB) [19]. The GFB not only functions as a mechanical barrier, preventing the entry of macromolecules into the urine, but also as an electrostatic barrier, preventing the passage of negatively charged substances [20]. There is a basis for interaction between endothelial cells and podocytes that is largely dependent on angiogenic molecules such as vascular endothelial growth factor (VEGF). If this signaling is interrupted, it may result in damage to endothelial cells or podocytes and disruption of glomerular filtration barrier integrity [21]. Therefore, maintaining normal interactions and signaling between endothelial cells and podocytes is crucial for preserving the integrity and function of the glomerular filtration barrier.

The NF-κB pathway, an essential controller in the inflammatory response, plays a central role in acute kidney injury (AKI) through its signaling pathways. Circulating pathogen-associated molecular patterns (PAMPs) such as endotoxin lipopolysaccharide (LPS) further activate NF-κB by activating toll-like receptors (TLRs) and myeloid differentiation 88 (MyD88) on renal tubular cells. This process induces the involvement of interleukin-1 (IL-1) receptor-associated kinase (IRAK) and tumor necrosis factor receptor-associated factor 6 (TRAF-6), leading to NF-κB dimerization (p65, p50) into the nucleus, which stimulates the transcription of inflammatory genes such as TNF-α and IL-6 [22–24], leading to the onset of an inflammatory response. In addition, nod-like receptor thermal protein domain associated protein 3 (NLRP3) inflammasome under TNF-α-mediated activation of Toll-like receptor 4 (TLR4) or tumor necrosis factor receptor (TNFR) also leads to an increase in the production of NLRP3, IL-1β, and IL-18 through the NF-κB signaling pathway, exacerbating the inflammatory response of the organism [25–27]. These findings reveal the mechanism by which the NF-κB signaling pathway induces and exacerbates the inflammatory response in AKI. The TLR4/MyD88/NLRP3 signaling pathway directly controls the production of cytokines that activate the inflammatory cascade response, and it has been implicated in the development of chronic diabetes [28] and related renal diseases as a novel therapeutic target.

**Fig. 1:**
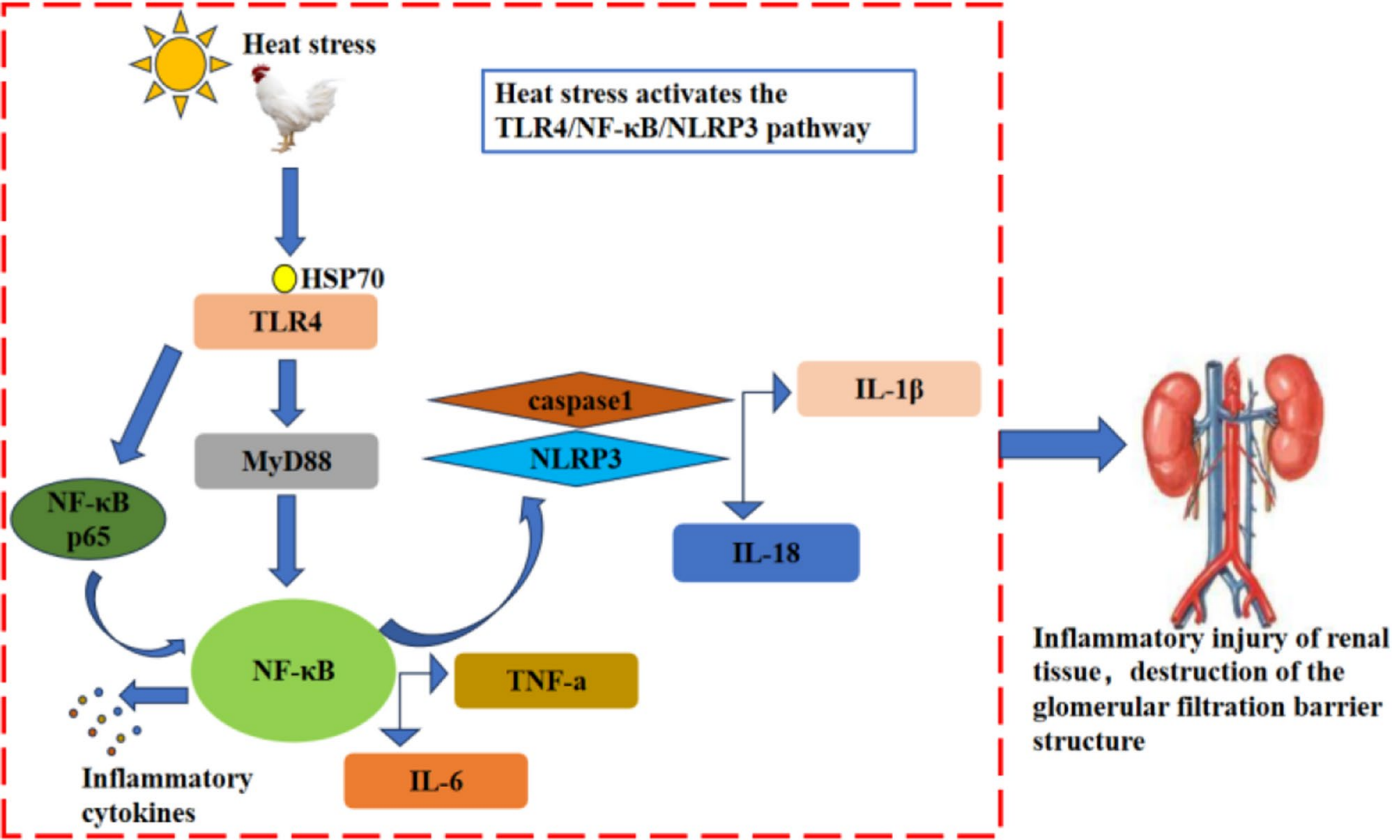
Mechanism of inflammatory damage in broiler kidney tissue due to heat stress. Note: Heat stress stimulates the organism to induce extracellular or extracellular HSP70 overexpression, HSP70 binds to TLR4 and activates TLR4, which activates downstream MyD88 to indirectly activate NF-κB or TLR4 activation can directly activate NF-κB, resulting in the release of inflammatory cytokines such as (TNF-α, IL-6) from NF-κB. The activation of NF-κB causes NLRP3 overexpression; NLRP3 activates downstream caspase-1 and promotes the release of IL-1β and IL-18, leading to an increased renal inflammatory response and an impaired filtration barrier

Global warming and rising temperatures, this high temperature environment has caused a huge impact on the livestock and poultry farming industry. Relevant scholars have found that heat stress adversely affects broiler kidneys, but there are fewer studies related to the impact on the glomerular filtration barrier, and the mechanism of glomerular filtration barrier damage caused by heat stress is not clear. Therefore, the objective of this work is to investigate the process of kidney injury induced by the activation of TLR4/NF-κB/NLRP3 signaling pathway by heat stress disrupting the filtration barrier in broiler chickens and to give a certain experimental basis for exploring the mitigation of the damages incurred as a result of heat stress to the livestock and poultry farming industry (Fig. 1).

## Results

### The impact of heat stress on production performance

Average daily weight gain, average daily feed consumption and feed-to-weight ratio were calculated by determining broilers’ body weight at 21 and 42 days of age, as indicated in Table 1. In comparison to the TN group, the body weight of broilers in the HS group was remarkably decreased and there was no significant difference in body weight at 21 days (*P* > 0.05); body weight at 42 days of age was very remarkably reduced (*P* < 0.01); average daily feed intake was very remarkably decreased (*P* < 0.01); daily weight gain was remarkably decreased (*P* < 0.05) and feed-to-meat ratio was remarkably elevated (*P* < 0.05) and these findings suggested that heat stress reduced the mean daily consumption of feed and the mean daily rise in weight collectively result in an elevation of the feed-to-weight ratio.

**Table 1:** The influence of heat stress on the growth performance of AA broilers

Items	Group		*P*-value
	TN	HS	
21-day-old weight/g	929.80 ± 58.75	928.40 ± 27.61	0.063
42-day-old weight/g	2724.80 ± 72.87	2237.60 ± 70.00	0.001
ADFI/(g/d)	85.48 ± 10.09	61.68 ± 7.16	0.000
ADG/(g/d)	60.00 ± 6.30	35.26 ± 4.62	0.003
F/G(%)	1.42 ± 0.33	1.75 ± 0.20	0.011

Note: TN denotes normal group; HS denotes heat stress group; data were presented as mean ± SD (*n* = 5), with *P* < 0.05 denoting significant difference and *P* > 0.05 denoting NS

### The impact of heat stress on serum pro-inflammatory cytokines and serum corticosterone in broilers

**Fig. 2:**
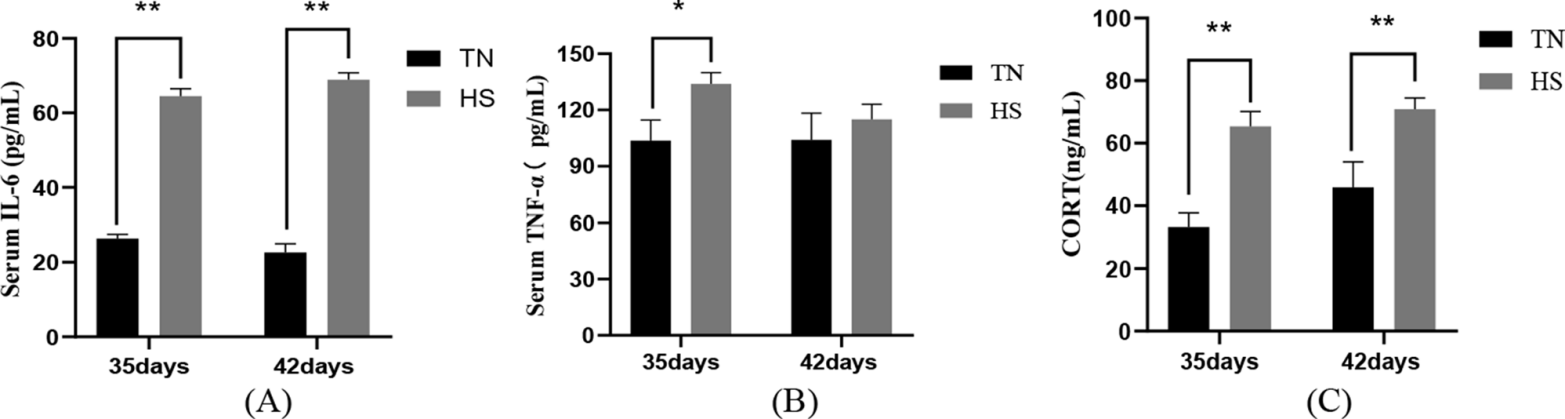
The impact of heat stress on serum IL-6 (**A**), TNF-α (**B**) and CORT (**C**) concentrations in broilers of different days of age. TN denotes the normal group; HS denotes the heat stress group; data were expressed as mean ± SD (*n* = 5). *P* < 0.01 indicates a highly significant difference, *P* < 0.05 indicates a significant difference and *P* > 0.05 indicates NS (∗*P* < 0.05, ∗∗*P* < 0.01)

The results of serum pro-inflammatory cytokines demonstrated that in comparison to the TN group, the serum IL-6 content was highly remarkably elevated (*P* < 0.01) and serum TNF-α content was remarkably elevated (*P* < 0.05) in the 35-day-old HS group and the IL-6 concentration was highly remarkably elevated (*P* < 0.01) in the 42-day-old HS group, but there was no notable disparity in the quantity of TNF-α present in the serum. In addition, the findings of serum corticosterone demonstrated that the serum CORT concentration was highly remarkably elevated (*P* < 0.01) in both 35- and 42-day-old groups in comparison to the TN group, indicating that heat stress caused inflammatory damage to the organism and hormone metabolism disorders in vivo, as shown in Fig. 2.

### The impact of heat stress on antioxidant-related indexes

**Fig. 3:**
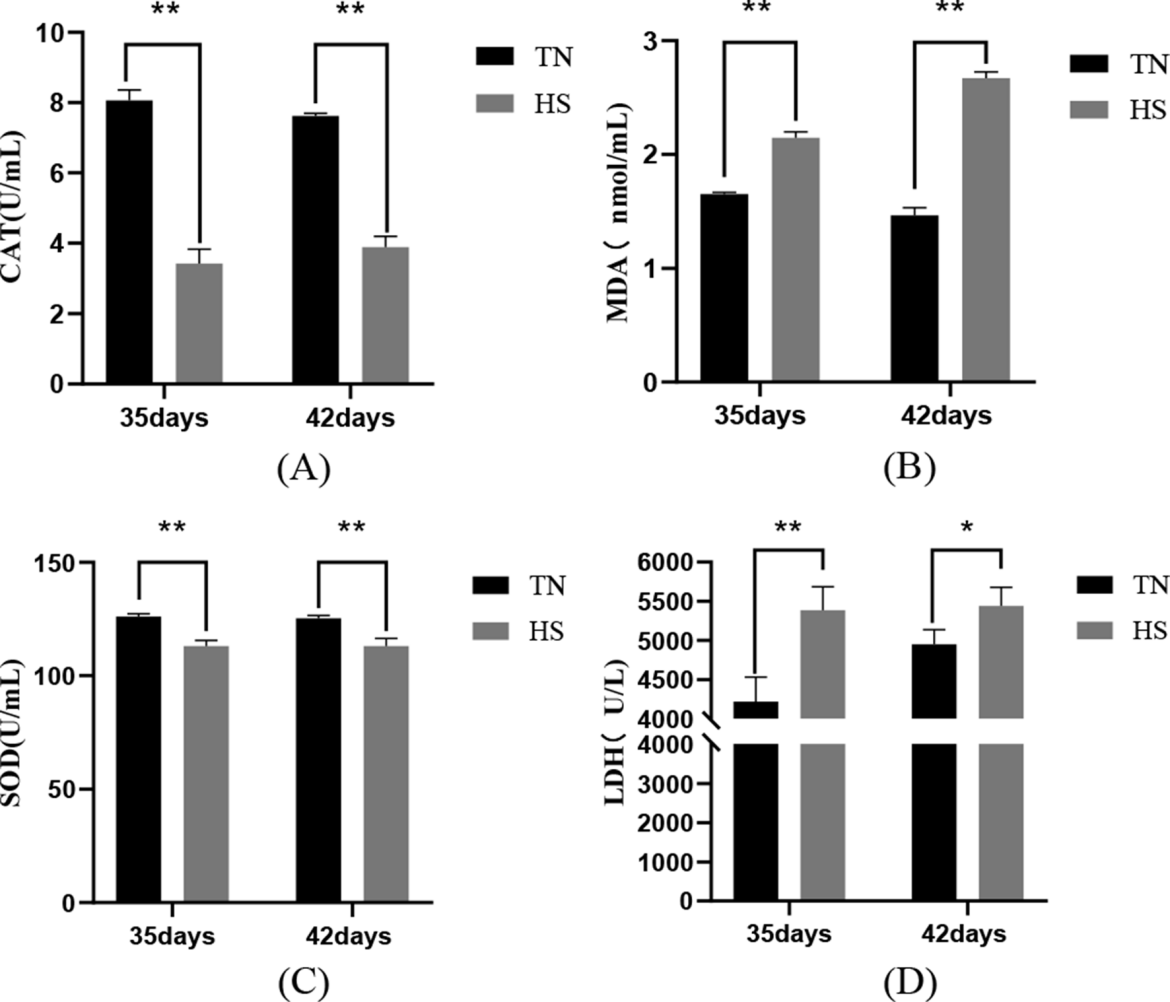
The impact of heat stress on antioxidant-related indices CAT (**A**), MDA (**B**), SOD (**C**) and LDH (**D**) in broilers of different ages. TN indicates the normal group; HS indicates the heat stress group; data were presented as mean ± SD (*n* = 5). *P* < 0.01 indicates a highly significant difference, *P* < 0.05 indicates a significant difference (∗*P* < 0.05, ∗∗*P* < 0.01)

The results of serum antioxidant-related indexes demonstrated that in comparison to the TN group, serum CAT and SOD activities of the 35-day-old HS group were extremely remarkably reduced (*P* < 0.01), the serum MDA content and LDH activity of the HS group were extremely remarkably elevated (*P* < 0.01); at 42 days old: serum CAT and SOD activities of the HS group were extremely remarkably reduced (*P* < 0.01), serum MDA level of the HS group was extremely remarkably elevated (*P* < 0.01) and serum LDH activity remarkably elevated (*P* < 0.05), these results demonstrated that heat stress leads to oxidative stress in the organism and aggravates oxidative damage in tissues and organs, as shown in Fig. 3.

### The impact of heat stress on renal function indexes of broilers

**Fig. 4:**
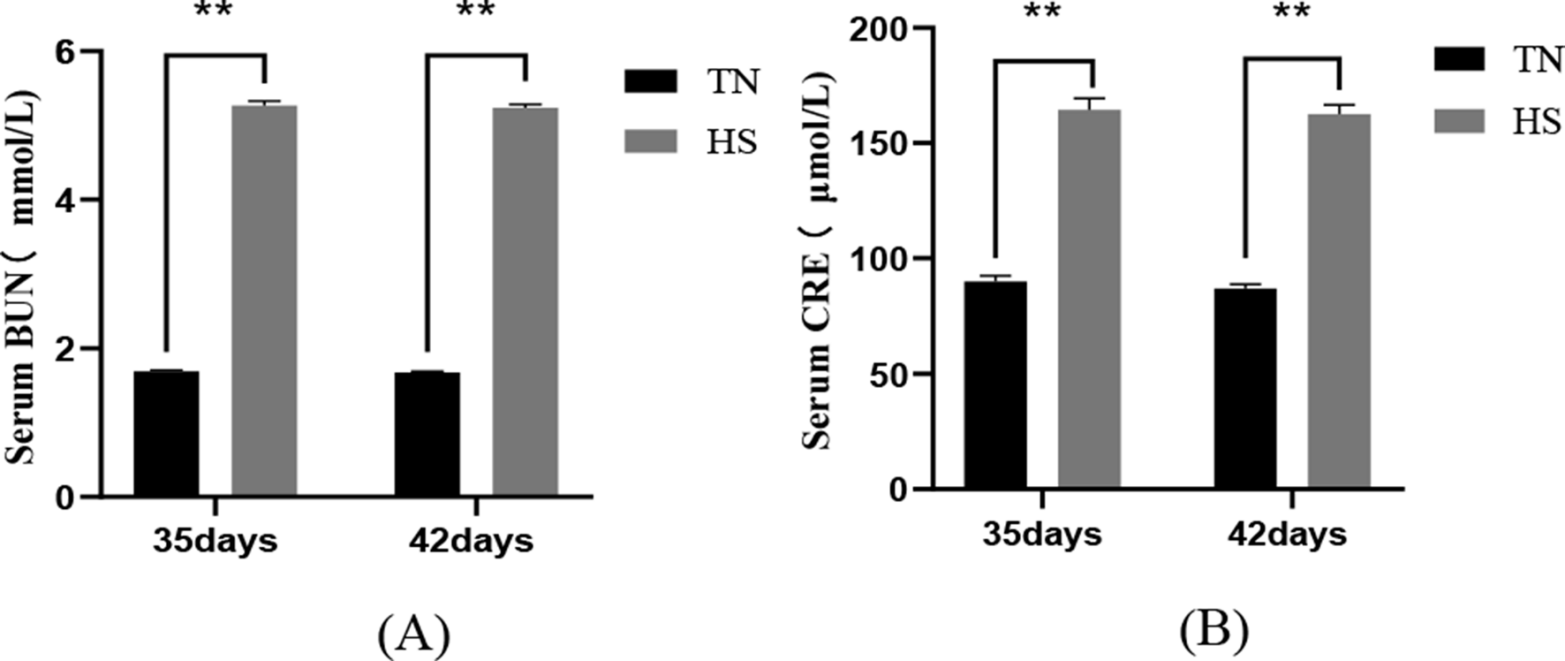
The impact of heat stress on the production of BUN (**A**) and CRE (**B**), indicators of renal function, in broilers of different days of age.TN denotes the normal group; HS denotes the heat stress group; data are presented as mean ± SD (*n* = 5). *P* < 0.01 indicates a highly significant difference, *P* < 0.05 indicates a significant difference (∗*P* < 0.05, ∗∗*P* < 0.01)

The results of renal function indexes demonstrated that the serum BUN and CRE content of broilers in the 35-day and 42-day HS group were extremely remarkably elevated (*P* < 0.01) in comparison to the TN group and these results indicated that heat stress led to impaired renal filtration function, as shown in Fig. 4.

### The impact of heat stress on serum ion concentrations

The results of serum ion concentrations demonstrated that in comparison to the TN group, the serum concentrations of Na^+^, K^+^, Cl^−^ and HCO_3_^−^ in the HS group all demonstrated a decreasing trend after heat stress, of which the difference in the concentration of Cl^−^ was not significant (*P* > 0.05). The decreasing trend of the concentrations of Na^+^, K^+^ and HCO_3_^−^ was highly significant at the age of 35 days (*P* < 0.01) but the decreasing trend of the concentrations of each ion at the age of 42 days was alleviated (*P* < 0.05). The findings indicated that the selective permeability of the glomerular filtration barrier to Na^+^, K^+^ and HCO_3_^−^ was altered by heat stress, as indicated in Table 2.

**Table 2:** The effect of heat stress on serum ion concentrations in AA broiler chickens of different days of age

Items		Group		*P*-value
		TN	HS	
Na^+^(mmol/L)	35days	148.77 ± 1.77^a^	142.18 ± 3.03^c^	0.004
	42days	148.19 ± 2.09^a^	139.40 ± 2.63^b^	0.012
K^+^(mmol/L)	35days	4.75 ± 0.39^a^	4.08 ± 0.42^c^	0.001
	42days	4.22 ± 0.75^a^	4.01 ± 0.49^b^	0.071
Cl^-^(mmol/L)	35days	101.05 ± 4.33	98.63 ± 1.02	0.437
	42days	97.89 ± 2.50	96.88 ± 1.78	0.605
HCO_3_^-^(mmol/L)	35days	21.25 ± 0.21^a^	18.61 ± 0.71^c^	0.001
	42days	21.65 ± 0.30^a^	19.74 ± 0.26^b^	0.016

Note: Distinct consecutive letters for the same ion concentration signify noteworthy disparities (*P* < 0.05), letters at distinct intervals suggest very substantial disparities (*P* < 0.01) and the same or absence of a letter denotes NS (*P* > 0.05)

### The impact of heat stress on the morphology and structure of the glomerular filtration barrier (histopathological observation)

**Fig. 5:**
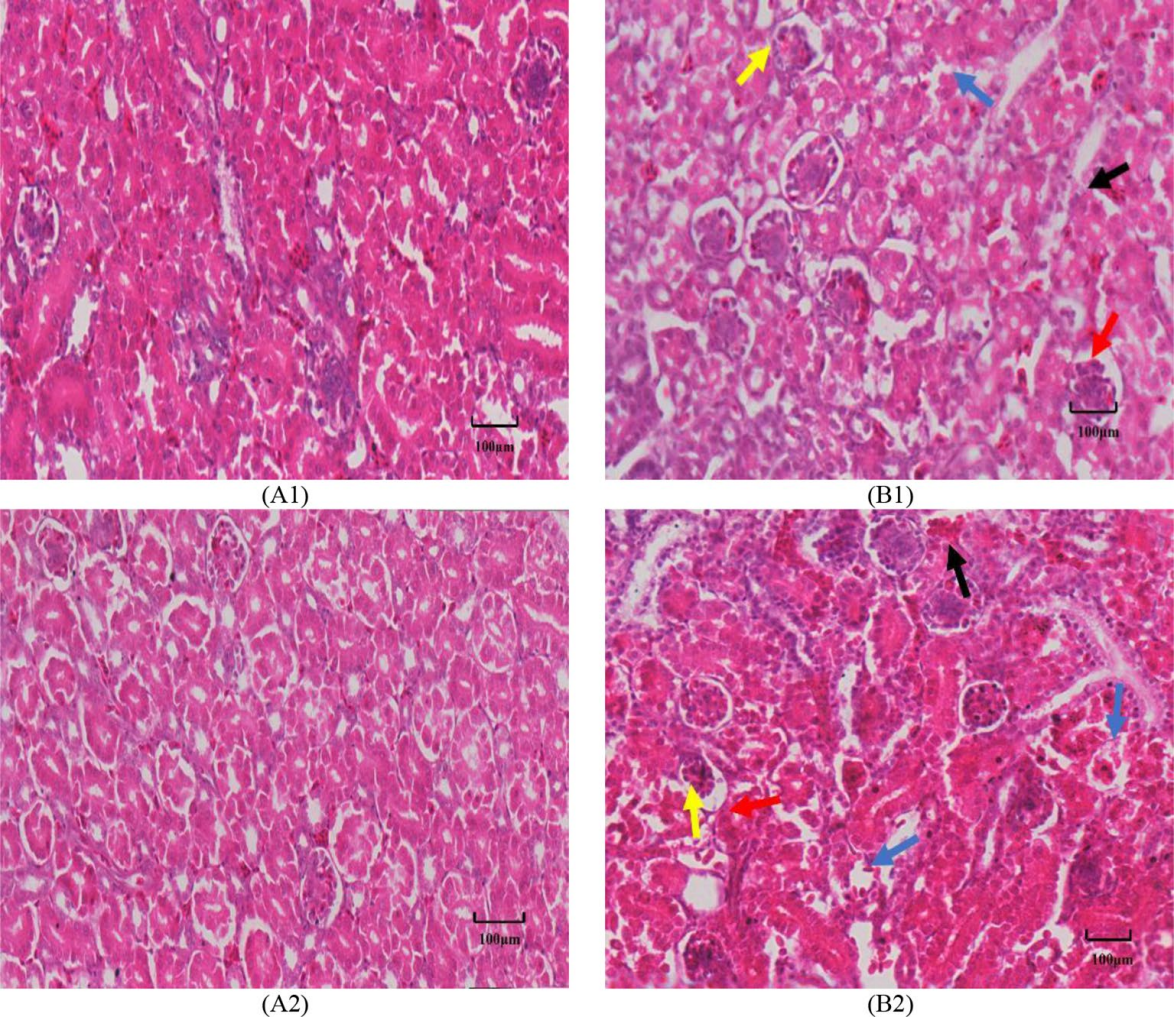
The impact of heat stress on histopathological changes of kidney in broilers of different ages (HE staining; scale bar: 20X, 100 μm). A1, 35-day-old TN group; B1, 35-day-old HS group; A2, 42-day-old TN group; B2, 42-day-old HS group. Note: blue arrows: renal tubules demonstrated granular and vacuolar degeneration, partial exfoliation of epithelial cells, rupture and necrosis; red arrows: basement membranes were bare; yellow arrows: part of the inner cells of the vascular glomeruli were infiltrated, atrophied and fragmented; black arrows: interstitial congestion and/or hemorrhage

Histopathological results demonstrated that in comparison to the TN group, the HS group demonstrated slight congestion and hemorrhage in some glomeruli and tubular interstitium, inflammatory cell infiltration, and fragmented nuclei formation in the HS group, slight thickening of the glomerular basement membrane, some of which appeared to be exposed; granular and vacuolar degeneration of tubular epithelium and epithelial cell exfoliation, rupture and necrosis of the epithelial cell; renal mesangial lymphocyte monocyte and neutrophil infiltration; angiosphere cell infiltration, some of which were atrophied and slightly fragmented. infiltration; infiltration of vascular glomerulocytes with partial atrophy and slight fragmentation, these findings suggest that heat stress induces some degree of harm to renal structures (Fig. 5).

### The impact of heat stress on the morphology and structure of the glomerular filtration barrier (transmission electron microscopy)

**Fig. 6:**
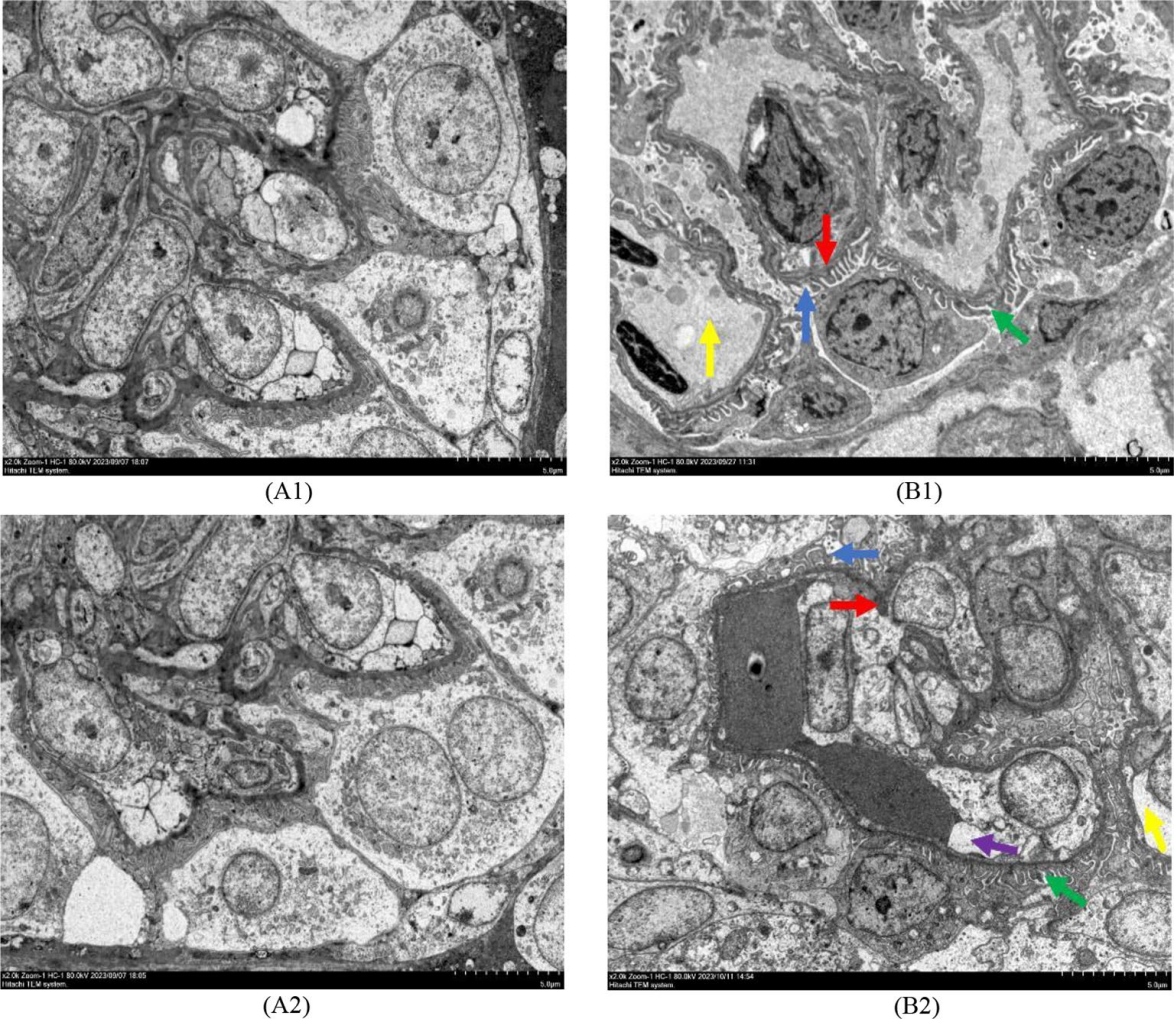
The impact of heat stress on the ultrastructure of glomerular filtration barrier in broiler chickens of different days of age (Transmission electron microscopy observation; Scale bar: 2000X, 5 μm). A1, 35-day-old TN group; B1, 35-day-old HS group; A2, 42-day-old TN group; B2, 42-day-old HS group Note: red arrows: thickening of the basilar membrane; dark blue arrows: disappearance of breaks in the peduncle and shortening of its length; yellow arrows: widening of the subendothelial pellucid zone; green clippings: narrowing of the lacunar membrane; purple arrows: gradual narrowing of the subendothelial pellucid zone from wide to narrow

Observation of the ultrastructure of the filtration barrier by transmission electron microscopy revealed that, in comparison to the TN group, the filtration barrier of broiler kidney in the HS group all demonstrated breakage and disappearance of podocyte peduncles, with a significant shortening of the length of the peduncle; diffuse thickening of the basement membrane, narrowing of the fissure membrane and a widening of the subendothelial hyaline zone; however, the situation was alleviated at the age of 42 days, e.g., the subendothelial hyaline zone was narrowed from wide to narrow (Fig. 6).

### The impact of heat stress on HSPs and VEGF mRNA expression in renal tissues

**Fig. 7:**
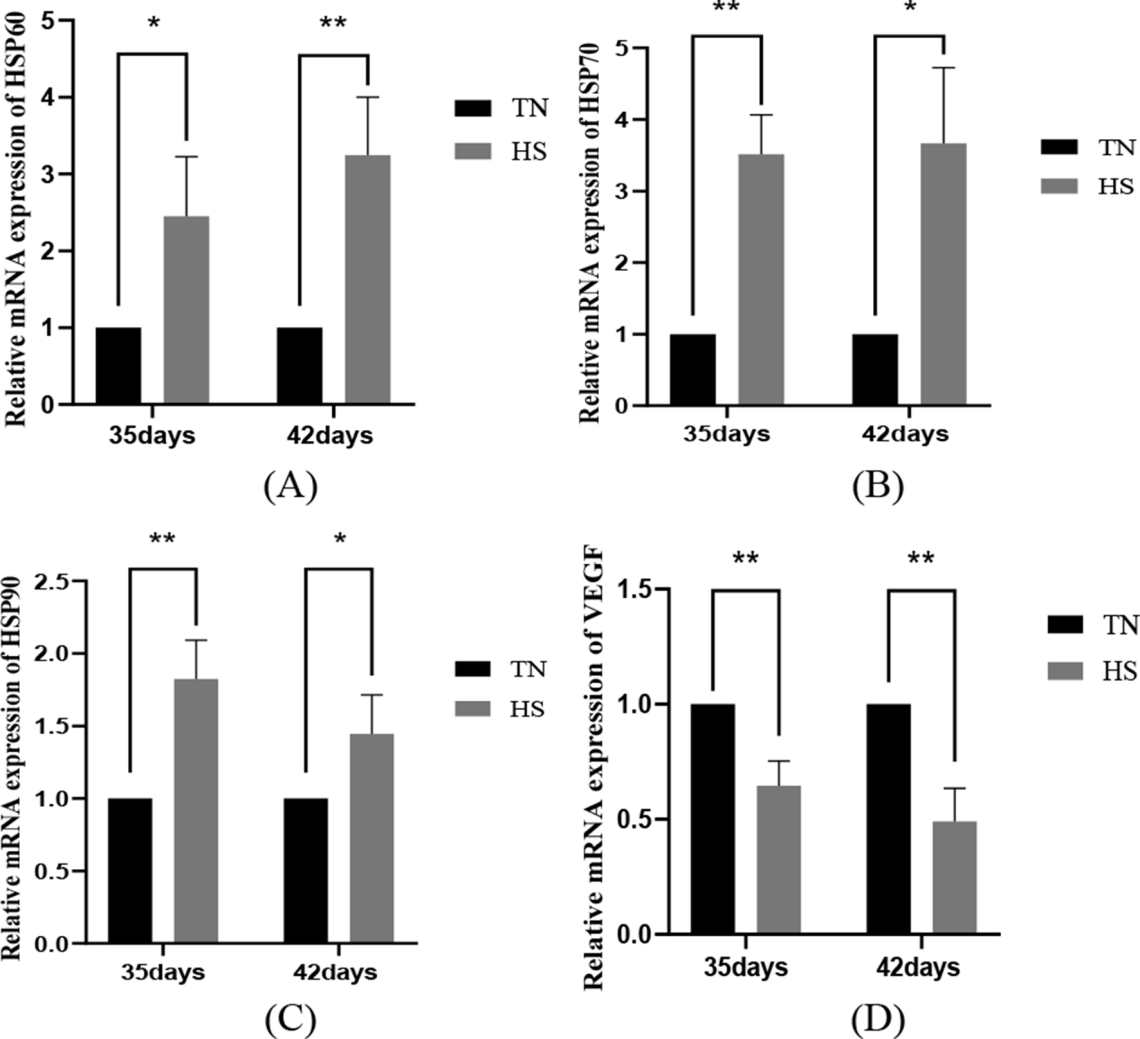
The impact of heat stress on the production of HSP60 (**A**), HSP70 (**B**), HSP90 (**C**) and VEGF (**D**) in kidney tissues of broilers of different ages. Data were shown as mean ± SD (*n* = 5). *P* < 0.01 indicates a highly significant difference, *P* < 0.05 indicates a significant difference and *P* > 0.05 indicates NS (∗*P* < 0.05, ∗∗*P* < 0.01)

Heat shock proteins are widely distributed in the kidney and play important roles at the same time. In comparison to the TN group, the heat stress results on the mRNA levels of HSPs in broiler kidney tissues showed that HSP60 mRNA was remarkably elevated in the HS group of broiler kidney tissues at the age of 35 days (*P* < 0.05), of which, HSP70 and HSP90 mRNA were highly remarkably elevated (*P* < 0.01). 42-day-old broiler kidney tissues of the HS group had remarkably elevated HSP70 and HSP90 mRNA (*P* < 0.05); among them, HSP60 mRNA was highly remarkably elevated in the HS group (*P* < 0.01). In addition, VEGF is crucial for maintaining the integrity and function of the glomerular filtration barrier, which safeguards the normal filtration function of the kidney by maintaining close interaction and signaling between endothelial cells and podocytes. The results of heat stress on VEGF mRNA expression in renal tissues demonstrated that VEGF mRNA in renal tissues of broiler chickens in the HS group was extremely remarkably reduced (*P* < 0.01) compared with that in the TN group. The findings reveal that heat stress induces an increase in heat shock proteins and a decrease in vascular endothelial growth factor in the body, leading to damage to endothelial cells or podocytes, which will disrupt the integrity of the filtration barrier and affect its filtration function, as shown in Fig. 7.

### The impact of heat stress on the activation of genes linked to TLR4/NF-κB/NLRP3 pathway in kidney tissues

**Fig. 8:**
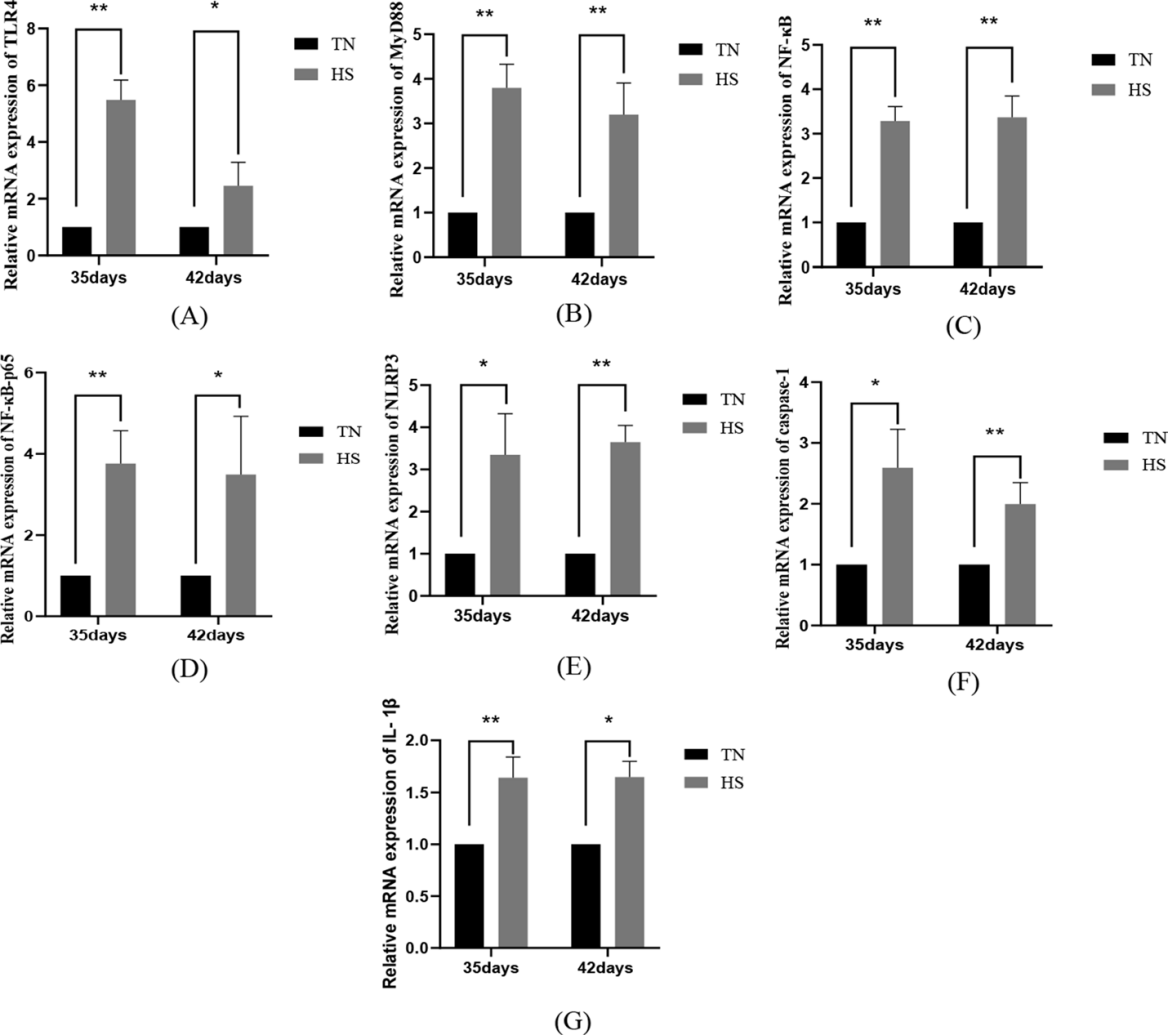
The impact of heat stress on mRNA activation of TLR4 (**A**), MyD88 (**B**), NF-κB (**C**), NF-κB-p65 (**D**), NLPR3 (**E**), caspase-1 (**F**) and IL-1β (**G**) in renal tissues of broilers of different days of age, with the data presented as mean ± SD (*n* = 5). *P* < 0.01 indicates a highly significant difference, *P* < 0.05 indicates a significant difference (∗*P* < 0.05, ∗∗*P* < 0.01)

The results of heat stress on the activation of genes linked to TLR4/NF-κB/NLRP3 pathway in renal tissues demonstrated that TLR4, MyD88, IL-1β, NF-κB and NF-κB-p65 mRNA were highly remarkably elevated in the HS group of renal tissues of broilers at the age of 35 days (*P* < 0.01) and NLPR3 and caspase-1 mRNA were highly significant elevated in the HS group of renal tissues (*P* < 0.05), when in comparison to that of the TN group; 42 days of age, the NLPR3 and caspase-1 mRNA were highly significant elevated (*P* < 0.05); MyD88, NLPR3, caspase-1 and NF-κB mRNA were highly remarkably elevated (*P* < 0.01), and TLR4, IL-1β and NF-κB-p65 mRNA was remarkably elevated (*P* < 0.05) in the HS group of kidney tissues of broilers at 42 days old, as shown in Fig. 8.

## Discussion

High-temperature weather often adversely affects the growth performance of broilers. Due to the special physiological structure of poultry, they rely on evaporation to dissipate heat, which greatly reduces the efficiency of heat dissipation, having the result of making broilers consume more water and less feed, which in turn reduces their production performance [29]. High-temperature environments can also lead to changes in serum hormone levels, which can trigger metabolic disorders and reduce growth rates. In addition, heat stress induces an accelerated gluconeogenic response, which speeds up the catabolic process of proteins and fats, while shortening the residence time of the feed in the digestive tract [30–32], reducing the feed conversion rate. The current investigation’s findings showed that heat stress resulted in remarkably elevated serum corticosterone levels, remarkably decreased mean daily weight gain and mean daily feed intake, and increased feed-to-weight ratio in broilers at 35 and 42 days of age, suggesting that heat stress leads to reduced growth performance in broilers.

Heat stress often triggers oxidative stress, leading to increased pro-inflammatory cytokines and oxidative damage. The persistence of oxidative stress leads to elevated inflammatory factors such as TNF-α and IL-6 in the blood, which in turn leads to organ damage and oxidative cellular injury [11, 33]. In a related study, it was found that after a period of heat stress, SOD, CAT and GSH-Px activities in the broiler chicks’ serum were reduced [34], and LDH activity and MDA content were increased [35–37]. The above results suggest that heat stress induces an imbalance in the redox response of the organism, exacerbates the inflammatory response, and leads to oxidative damage in renal tissues. The current investigation’s findings demonstrated that heat stress significantly reduced SOD and CAT activities and significantly increased LDH activity and MDA content, antioxidant indexes, in broilers at 35 and 42 days old, suggesting that heat stress triggers oxidative damage in the organism.

The kidney is an important organ for urine production and excretion in animals, responsible for retaining nutrients and excreting wastes, as well as controlling the body’s water, electrolyte and acid-base balance [38]. Heat stress leads to an increase in endotoxin in the body, and the kidneys are the main target of endotoxin attack, often leading to inflammatory kidney injury in the process [39]. Chronic heat stress significantly increased serum urea nitrogen and serum creatinine levels, indicating renal insufficiency [40, 41]. It has been shown that enhanced evaporation and heat dissipation in broilers under heat stress prompts the kidneys to excrete more Na^+^, K^+^ and other alkaline cations, which further accelerates the loss of these electrolytes from the blood, and increases electrolytes in other vital organs. Heat stress also elevates endotoxin levels in the blood, which in turn stimulates increased secretion of aldosterone, which serves to retain Na^+^ and excrete K^+^, so that the Na^+^ content decreases markedly in the early phases of heat stress but slows down in the later phases, and these changes together lead to severe kidney damage [42–44]. The current study’s findings demonstrated that the HS group’s serum BUN and CRE levels were remarkably elevated than those of the TN group. This change reflected the decrease in glomerular filtration rate and damage to the filtration barrier. In addition, serum Na^+^, K^+^, Cl^−^ and HCO_3_^−^ concentrations in broilers demonstrated a decreasing trend after heat stress, but this trend was all alleviated at 42 days of age. These changes may be the reason why the organism promotes the metabolism of substances in an effort to keep the internal environment stable.

Related studies have found that the endothelial surface of normal glomeruli is flat and contains many transcellular septal pores. The filtration rate per unit area of the glomerular capillary membrane is strongly influenced by the diameter of the septal pores of the glomerular capillary endothelial cells, the thickness of the basement membrane, and the condition of the peduncle. Serum creatinine concentration was positively correlated with basement membrane thickness and changes in podocyte peduncles; serum creatinine concentration was negatively correlated with glomerular capillary endothelial cell pore diameter, glomerular capillary surface area density, and the percentage of glomerular capillary lumen size [45], suggesting that the glomerular filtration barrier will be affected accordingly when creatinine concentration changes. Heat stress disrupts the structural integrity of the kidney, leading to a compromised filtration barrier. Renal injury is exacerbated by shedding of renal tubular epithelial cells, reduction in the diameter of vascular glomeruli, and infiltration of large numbers of inflammatory cells [46]. The current investigation’s findings demonstrated that HE staining results at 35 and 42 days of age demonstrated varying degrees of vascular bulb reduction and inflammatory cell infiltration in the renal tissues of both HS groups at both days of age. Transmission electron microscopy results demonstrated that the foot cell peduncle in the glomerular filtration barrier of HS groups at 35 and 42 days of age demonstrated different degrees of atrophy and breakage or disappearance, and thickening of the basement membrane, etc., but the situation was alleviated at the age of 42 days, which may be related to the activation of the thermal protection mechanism of the animal’s organism.

Heat shock proteins can enhance cell tolerance and survival under certain conditions, but overexpression of heat shock proteins is one of the reasons for the aggravation of heat stress [47]. HSP70 and HSP90 are important for the maintenance of organismal metabolism and organ integrity, and chronic heat stress leads to the concentrations of HSP60, HSP70 and HSP90 mRNAs to rise noticeably in the muscle, heart and brain tissues of broiler chickens [32, 48], resulting in altered organism metabolism and organ integrity. HSP70 can bind to TLR4 and activate an inflammatory reaction, stimulating the synthesis and secretion of pro-inflammatory cytokines in the animal organism. Yang, W.C. found that when heat stress caused acute lung injury in rats, lung tissue vascular endothelial growth factor-A (VEGF-A) levels were reduced compared with the blank group [49]. Ginkgo bilabial extract (GBE)can exert a protective impact against hypoxic-ischemic renal injury in neonatal rats by increasing the activation of VEGF, suggesting that a decrease in vascular endothelial factors leads to renal injury [50]. The findings of this investigation revealed that HSP60, HSP70 and HSP90 mRNA activation were increased and VEGF mRNA activation was decreased in renal tissues of 35- and 42-day-old broilers, indicating that the degree of thermal injury in the kidney tissues escalated as the length of heat exposure increased, and that the filtration barrier was severely affected.

Heat stress leading to renal dysfunction is often accompanied by an inflammatory response. Inflammatory vesicles play a crucial role in innate immunity. activation of TLR4 stimulates relevant inflammatory signaling pathways. Evidence has demonstrated that heat stress remarkably upregulates the activation of TLR4 mRNA [39], which in turn binds TLR4 to MyD88, activates NF-κB and promotes transcription of inflammatory cytokines. This TLR4/MyD88/NF-κB signaling pathway directly regulates cytokines that stimulate the inflammatory cascade response [51, 52]. In addition, heat eat stress activates NLRP3/NF-κB inflammatory vesicles [29], enhances IL-6 and TNF-α signaling, and triggers IL-1β secretion [53, 54], thereby exacerbating the inflammatory response that leads to organismal. High activation of NLRP3 is associated with a reduced density of podocytes, glomerular sclerosis and volume increase in human glomeruli, and also triggers an inflammatory condition that thereby mediates kidney injury [55]. The findings found that heat stress increased the serum quantity of IL-6 and TNF-α and up-regulated the activation of TLR4/MyD88/NLRP3 signaling pathway-related genes in renal tissues of broilers at 35 and 42 days of age, but the up-regulation trend of serum inflammatory factors and signaling pathway-related genes was remarkably reduced at 42 days of age. These results indicate that heat stress can trigger the TLR4/MyD88/NLRP3 signaling pathway, leading to disruption to renal filtration barrier structure and further aggravating renal inflammatory injury. With the extension of heat stress, the degree of renal thermal injury is alleviated, which may be related to the thermotolerance of animals.

## Conclusion

The research study found that heat stress had a detrimental impact on the production performance of broiler chickens. Heat stress led to a decrease in average daily feed intake and daily weight gain, an increase in feed-to-meat ratio and a decrease in broiler performance. It resulted in altered serum antioxidant indices and exacerbated oxidative damage to renal tissues. It also resulted in an increased infiltration of inflammatory cells in kidney tissues, promoted the release of renal inflammatory factors, and contributed to the aggravation of renal inflammatory injury. Heat stress aggravated the inflammatory injury of renal tissues and damaged the glomerular filtration barrier structure through the activation of the TLR4/NF-κB/NLRP3 pathway, but the degree of injury varied among broilers of different days of age. With the prolongation of heat stress, renal tissue injury was aggravated, and the morphological structure and function of the filtration barrier were impaired, but the difference in activation of related genes in renal tissues of broilers at the age of 42 days was remarkably reduced. This may be due to the fact that broilers are a gradual adaptation process to heat stress, and the impact of heat adaptation is gradually obvious in the later stage, further slowing down the aggravation of heat injury, but relying on self-regulation alone, the protective the impact of heat adaptation against glomerular filtration barrier injury is not good. Therefore, the prevention of heat stress injury to livestock and poultry in large-scale broiler farming is an urgent problem to be solved. According to the findings of this paper, it is evident that heat stress harms renal tissue and glomerular filtration barrier injury in broilers through inflammatory damage. Therefore, in addition to improving environmental conditions, exploring the effective heat stress injury inhibiting drugs becomes a potential way to mitigate heat stress injury. These findings are of great significance in exploring the experimental basis for mitigating the glomerular filtration barrier damage in broilers under high-temperature conditions. Additionally, it aids in mitigating the economic damages resulting from heat stress in actual production.

## Materials and methods

### Test animals and experimental design

All experimental animals in our study were purchased from Luoyang Yiluoquan Agricultural Science and Technology Co. One hundred and twenty 1-day-old Arbor Acres (AA) male broilers were acquired from a hatchery operated for commercial purposes and were given in a temperature-controlled climate chamber at 35 °C, with relative humidity controlled between 45% and 55%, following the AA broiler rearing standards. With the growth of age, the ambient temperature was gradually adjusted from 35℃ to 23 ± 1℃. 100 birds with similar body weights were selected at 21 days of age and randomly divided into a normal group (TN group) and heat stress group (HS group), with 5 replicates (cages)/groups and 10 birds/replicates, and the of the TN group temperature maintained at 23 ± 1℃ to ensure sufficient feed and water. The temperature of HS group was maintained at 35 ± 1℃, the heat stress time was from 9:00 to 19:00, high temperature for 10 h per day, give sufficient feed and water, 19:00 to the next day 9:00 temperature maintained at 23 ± 1℃. Multi-layer cages were used to keep the chickens free to feed and drink throughout the feeding process, to minimize environmental stress, and to keep the test broilers in a relatively stable and suitable environment.

### Sample collection

When the broilers reached 35 and 42 days of age, one broiler from each replicate was randomly selected from each replicate cage for blood collection from the wing vein, and then euthanized by injection of 5% pentobarbital (at a dose of not less than 250 mg/kg) until the chickens were completely devoid of vital signs. Then the kidneys were removed and rinsed with saline, and the tissues were equally divided into three portions, and one portion was preserved in 10% formalin solution for hematoxylin-eosin (HE) staining for histopathological analysis; One copy was put into a tube of electron microscopy fixative and stored at -20℃ for electron microscopy to observe the ultrastructural changes of tissues; the last copy was put into another clean EP tube and stored at ultra-low temperature (-80℃) until ready for spare use.

### Growth performance

From 9:00 a.m. on day 21, body weight and amount of food consumed by broiler chickens 21 to 42 days of age were recorded, average daily feed intake (ADFI), average daily gain (ADG) and feed-to-weight ratio (F/G) were calculated. where ADG is total weight gain separated by the number of broilers multiplied by days, ADFI is total feed intake split by the number of broilers multiplied by days, and F/G is feed intake divided by weight gain multiplied by 100%.

### Measurement of pro-inflammatory cytokines and corticosterone in serum

At 35 and 42 days of age, blood was collected from broilers with appropriate body weight selected from each replicate and serum was separated. The OD values of pro-inflammatory cytokines IL-6 and TNF-α in serum were measured by the wavelength at 450 nm and the concentrations were calculated using ELISA kits produced by Wuhan Doctoral Biotechnology Co.

At 35 and 42 days of age, blood was collected from broilers with appropriate body weight selected from each replicate and serum was separated. The absorbance (OD value) at a wavelength of 450 nm was read on an enzyme labeling instrument (Shanghai Deacon Laboratory Equipment Co., Ltd.) using the Chicken Corticosterone (CORT) ELISA kit from Shanghai Enzyme-linked Biotechnology Co. The standard curve equation was established using four-parameter logarithmic curve fitting, and the concentration of the sample was calculated from the OD value of the sample.

### Antioxidant-related indicators

The serum of broilers to be tested was randomly selected from replicate groups at 35 and 42 days of age. Superoxide dismutase, catalase, lactate dehydrogenase activities and malondialdehyde content were determined according to the instructions using kits provided by Nanjing Jiancheng Bioengineering Institute.

First, in the determination of chicken serum superoxide dismutase concentration, the WST-1 method was used, following the operational guidelines, and the OD values were measured through the wavelength of 450 ± 10 nm to calculate the superoxide dismutase concentration in each group.

Then, in the determination of chicken serum catalase concentration, the ammonium molybdate method was used, following the operational guidelines, and the OD values were measured through the wavelength of 405 nm to calculate the concentration of catalase in each group.

Second, in determining the concentration of chicken serum lactate dehydrogenase, the microplate method was used, following the operational guidelines, and the OD values were measured through a wavelength of 450 nm to calculate the concentration of lactate dehydrogenase in each group.

Last, for the determination of chicken serum malondialdehyde content, the TBA method was used, following the operational guidelines, and the OD values were measured through the wavelength of 532 nm to calculate the malondialdehyde content of each group.

### Renal function indices

Sera to be tested were randomly selected from broilers in duplicate groups at 35 and 42 days of age. The urea nitrogen and creatinine contents were determined according to the instructions using the kits provided by Nanjing Jiancheng Bioengineering Institute.

Firstly, in the determination of urea nitrogen content in chicken serum, the urease method kit was used, and the operating instructions were followed to measure the OD value through the wavelength of 640 nm, and then calculate the urea nitrogen content of each group. According to the Nanjing Jiancheng Institute of Biological Engineering, the normal chicken serum urea nitrogen concentration should be 3.956 ± 2.294 mmol/L, and this range can be used as a basis for assessing whether the assay results are normal or not.

Secondly, when detecting creatinine content in serum, the microplate assay kit was used and operated in strict accordance with the operating instructions. The absorbance values A1 and A2 of each well were measured by 546 nm wavelength, and the creatinine content of each well was calculated accordingly. According to the data provided by the Nanjing Institute of Biological Engineering, China, the concentration range of normal chicken serum creatinine should be 88.164 ± 14.757 mmol/L, and this data can be used as a reference for judging whether the test results are normal or not.

Finally, serum ion concentration was determined by using Beijing Shengshi Dongtang Technology Co., Ltd (DOTOP-8018) automatic biochemical analyzer to determine serum Na^+^, K^+^, Cl^−^ and HCO_3_^−^ content.

### HE stained section preparation

Kidney tissues were dehydrated, transparent, dipped in wax, and embedded after fixation with paraformaldehyde, and sections of 4 ~ 6 μm thickness were prepared using a slicer (Leica Instruments GmbH, Germany). Hematoxylin-eosin staining was performed, and the morphological features of kidney tissues were observed under a light microscope (Olympus, Japan).

### Preparation of electron microscope sections

Kidney tissues less than 1 mm³ were selected and taken, fixed overnight with 3% glutaraldehyde, rinsed with phosphate buffer, re-fixed with 1% osmium fixative, rinsed again, dehydrated, embedded, cured, trimmed, sliced with ultrathin microtome, stained and observed.

### Real-time quantitative polymerase chain reaction assay

Tissue samples were removed from − 80 °C refrigerator, the production of HSP60, HSP70, HSP90, VEGF, TLR4, MYD88, NF-κB, NF-κB-p65, NLRP3, caspase-1, IL-1β in kidney tissues was detected by qPCR. Total RNA from kidney tissues was extracted using TRIzol reagent and the concentration was subsequently adjusted to 1000ng/µL using a spectrophotometer. The reverse transcription was conducted using the Hiscript III QRT SuperMix kit from Vazyme Biotech Co., located in Nanjing, China. In accordance with the manufacturer’s instructions. The quantitative PCR was performed using a Sequence Detection System (Thermo Fisher Science, Boston, MA) with a reaction volume of 20µL. The primer sequences listed in Table 3 were used to select β-actin as a control. The PCR program consisted of 95 °C denaturation for 30s, 95 °C denaturation for 10s, annealing at 60 °C for 30s, extension at 95 °C for 15s, extension at 60 °C for 60s and 95 °C extension for 15s for a total of 40 cycles. The relative transcript levels of each group of target genes were calculated by 2^−∆∆Ct^.

**Table 3:** Sequence information of oligonucleotide primers

Gene	Sequences(5’to3’)
β-actin F	CCGCTCTATGAAGGCTACGC
β-actin R	CTCTCGGCTGTGGTGGTGAA
HSP60 F	TGATGCGATGCTTGGGGAAT
HSP60 R	TCAGAGCCGTTCTCACAACC
HSP70 F	TGTGGCCTTCACCGATACAG
HSP70 R	TGGGGTCATCATACTTGCGG
HSP90 F	GGACCAACCAATGGAGGAGG
HSP90 R	TGCTCGGGTCAGTCAAACTC
VEGF F	ACAAGAAAATCACTGTGAGCCT
VEGF R	TGCTCACCGTCTCGGTTTTT
TLR4 F	CCAAACACCACCCTGGACTT
TLR4 R	CCATGGAAGGCTGCTAGACC
MYD88 F	GAGGGATGATCCGTATGGGC
MYD88 R	ACACGTTCCTGGCAAGACAT
NF-κB F	ACACCACTGGATATGGCAGC
NF-κB R	TCTTGCTTGGATCAGGCGTT
NF-κB p65 F	GGATTCCGGGCAGTGACG
NF-κB p65 R	CACGGCGCGCTAAAGTAAAG
NLRP3 F	GGTTTACCAGGGGAAATGAGG
NLRP3 R	TTGTGCTTCCAGATGCCGT
caspase-1 F	GATACGTGACTCCATCGACCC
caspase-1 R	CTTCTTCAGCATTGTAGTCC
IL-1β F	GCCTGCAGAAGAAGCCTCG
IL-1β R	AAGGACTGTGAGCGGGTGTA

### Statistics

Independent samples t-test in statistical software SPSS26 were used. Data were presented as mean ± standard deviation. Compared with the TN group, **P < 0.01 indicates an extremely significant distinction, *P < 0.05 indicates an important distinction, whereas *P* > 0.05 indicates NS.

## Data Availability

Data provided in manuscript documents.
